# The Book World of Medicine and Science

**Published:** 1895-02-02

**Authors:** 


### THE BOOK WORLD OF MEDICINE AND SCIENCE.

SPINAL CURVATURE AND AWKWARD DEPORTMENT: THEIR CAUSES AND PREVENTION IN CHILDREN. By Dr. GEORGE MULLER, Professor of Medicine and Orthopædics, Berlin. Translated by RICHARD GREEN, F.R.C.P., Edin. (Scientific Press, Limited, 428, Strand. 1894. 8vo., 88 pp., 22 illustrations, 2s. 6d.)

This little book is compiled not so much for practitioners as for parents and pedagogues, for whom a practical work from which technicalities have been eliminated as far as possible meets an undoubted want. It deals mainly with the causes and prevention of spinal curvature, for the development of which unsightly and often health-endangering affection the ignorance of the lay public engaged in the training of children is so largely responsible. The scope of the book may be best explained by the following extract from Dr. Müller's preface. " . . . . the physician is powerless to do much in the direction of preventing and overcoming mischievous complications in the development of children without the co-operation of two powerful allies, the parent and the teacher, for whose benefit I have dealt with matters that are well enough known to the profession"; to which Dr. Green truly adds, "It will aid the former (the parents) in steering clear of the primary causes of these spinal deformities, and in obtaining knowledge sufficient to detect slight deformities from the normal, at a stage too when treatment may be efficacious." A few extracts from the book itself may be cited. " . . . . the expression 'the child will grow out of it' has become a *terminus technicus*. Let me take this opportunity of explaining that even the smallest curvature of the spine will not grow of its own accord, and will not correct itself without aid." Perhaps the most important, because the most generally adopted evils that the author alludes to, are the faulty positions assumed in reading, in standing, and especially in writing. Every orthopædic surgeon recognises this latter as one of the most frequent causes of curvature; it is scarcely credible that the harmful, and at the same time often illegible, sprawling, spider-like, "sloping hand" should have obtained so long, for it has nothing whatever to recommend it. " . . . . the child holds her head straight, but pushes the whole of her body over to one side, that is to say as far as her neighbour will allow her . . . . the spine thus makes a turn to right or left, while the legs and pelvis remain unchanged." Directions are given as to carrying weights, school satchels, arrangement of school benches, and clothing, with special reference to corsets, boots, &c. Considerable space is devoted to suitable free exercises, and to the advantages of judicious massage and kinetics, which perhaps appeal more to medical than "lay" readers. The book is profusely illustrated principally from photographs.

